# Low prevalence of significant carotid artery disease in Iranian patients undergoing elective coronary artery bypass

**DOI:** 10.1186/1476-7120-5-3

**Published:** 2007-01-10

**Authors:** Mohammad K Tarzamni, Abbas Afrasyabi, Mehdi Farhoodi, Fatemeh Karimi, Sara Farhang

**Affiliations:** 1 Associate professor of Radiology, Department of Radiology, Tabriz university of medical sciences, Tabriz, Iran; 2 Professor of Cardiothoracic surgery, department of cardiothoracic surgery, Tabriz University of medical sciences, Tabriz, Iran; 3 Associate professor Neurology, Neurology department, Tabriz University of medical sciences, Tabriz, Iran; 4 Radiologist, Department of Radiology, Tabriz University of medical sciences, Tabriz, Iran; 5 General Practitioner, Department of Radiology, Tabriz University of medical sciences, Tabriz, Iran

## Abstract

**Background:**

Coronary artery bypass grafting ranks as one of the most frequent operations worldwide. The presence of carotid artery stenosis may increase the stroke rate in the perioperative period. Routine preoperative noninvasive assessment of the carotid arteries are recommended in many institutions to reduce the stroke rate.

**Methods:**

271 consecutive patients undergoing coronary artery bypass grafting at Shaheed Madani hospital of Tabriz, Iran (age, 58.5 Y; 73.1% male) underwent preoperative ultrasonography for assessment of carotid artery wall thickness.

**Results:**

Plaque in right common, left common, right internal and left internal carotid arteries was detected in 4.8%, 7.4%, 43.2% and 42.1% of patients respectively. 5 patients (1.8%) had significant (<50%) and 3 (1.1%) patients had critical (<70%) stenosis in internal carotid arteries. Plaque formation in common carotid was not significantly different between two genders but the stenosis of left internal carotid was more frequently seen among men. Patients with plaques in right or left internal carotid arteries were significantly older.

**Conclusion:**

Consecutive Iranian patients undergoing elective coronary artery bypass surgery show a very low prevalence of significant carotid artery disease.

## Background

Coronary artery bypass grafting (CABG) ranking as one of the most frequent operations worldwide, decreases the morbidity related to unstable angina and mortality in certain patients with chronic stable angina [1]. However CABG is associated with adverse neurological complications of which postoperative stroke is the most devastating.

Cross-sectional studies have reported association between intima media thickness (IMT) and cardiovascular risk factors [2,3], prevalent cardiovascular disease [4-6], peripheral atherosclerosis and stroke [7]. Carotid endarterectomy (CEA) has been performed in patients undergoing CAB in a staged or concomitant fashion resulting in more than 100 published studies.

Previous studies have attempted to identify clinical predictors of stroke after CABG.

Several studies have shown that increased IMT confers risk of suture stroke [8,9]However some indicate no increased risk [10,11] and suggeste the clinical data as stronger prognostic indicators than preoperative common carotid artery (CCA) -IMT.

The prevalence of postoperative cerebrovascular disease seems to be lower in Iran as reported by Sadeghi et al [12]. We investigated the state of coronary artery disease in Iranian patients undergoing elective CABG hypothesing that carotid artery disease may be less frequent because of their younger age while undergoing operation.

## Methods

The subjects of his study were enrolled from consecutive patients of the Shaheed Madani university hospital (Tabriz, north west of Iran), who have been referred for elective CABG between January 2005 to February 2006.

Patients who had carotid endartrectomy and Patients with collagen – vascular diseases were excluded. The base- line examination included a medical history and laboratory testing. The Carotid arteries were evaluated with high – resolution B-mode ultrasonogaphy with a 7.5- MHz linear – array transducer (EUB- 525 Hitachi) to evaluate the presence and site of plaques and to qualify the degree of stenosis. The protocol involved scanning the CCAs, the carotid bifurcations, and the origins of the internal and external arteries. Patients were examined in a supine position, with the head in the axis of the body slightly tilted to the either side. Carotid intima media thickness (IMT) was measured as the distance between two parallel echogenic lines corresponding to the blood- intima and media- adventitia interfaces.

The common and internal carotid arteries were scanned cross- sectionally and longitudinally. All ultrasound measurements were recorded in end diastole and were measured by the same investigator blinded to patients clinical state. Interobserver reproducibility has been reported in other study using the same method for IMT evaluation[13]. Carotid artery stenosis and peak systolic flow were graded using the criteria of the society of Radiologists in Ultrasound Consensus Criteria [14].

Data were analyzed using the SPSS package (version 13). Continuous variables (CIMT, age) were compared using the student's t test. Correlations between nominal variables were analyzed using chi- square test. Statistical testing was done at the 2- tailed level of 0.05.

## Results

A total of 271 consequent patients undergoing elective CABG were included in this study. The mean age (± SD) of the patients was 58.5 ± 9.4 years. Baseline clinical characteristics of the patients are presented in Table 1.

**Table 1:** clinical base line characteristics of study population, undergoing elective CABG

	N (%)
Age (%>60 yrs)	118 (43.5)
Sex (%male)	198 (73.1)
Diabetes mellitus	76 (28.0)
Elective CABG	271 (100)
Previous stroke	7 (2.6)
Smoking	100 (36.9)
Smoking (> 10/day)	91 (33.5)

Diabetes mellitus was more common among women (p = 0.001) which had a higher mean of age (p = 0.020), while smoking was more common among men (p = 0.010) and was not related to age of the patients.

**Figure 1:**
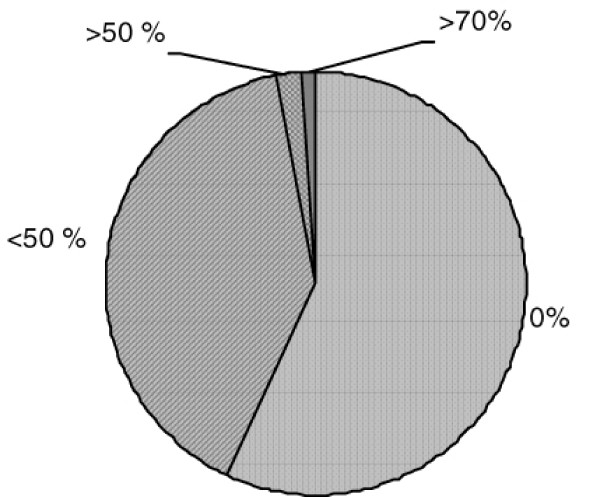
**Number of patients undergoing elective CABG according to the degree of observed stenosis of internal carotid arteries**.

A history of cerebro-vascular accident (CVA) was related to higher grade of stenosis in common carotid which had a higher peak systolic flow as well. Plaque formation in common and internal carotid arteries did not seem to be related to smoking nor to diabetes mellitus in the study population. Overall numbers of patients according to the degree of observed stenosis of internal carotid arteries are presented in figure 1.

Results of preoperative carotid study of patients are presented in Table 2 and Table 3. The peak systolic flow in both right and left carotid arteries was higher in advanced stages of the stenosis (p < 0.0005).

**Table 2:** Characteristics of 271 patents undergoing elective CABG, Number of patients with plaque in carotid arteries categorized by grade of the stenosis.

	Grade of stenosis				
	
	Normal	< 50%	50 – 69%	70 %- near occlusion	occlusion
Right common carotid artery	258	13	-	-	-
Left common carotid artery	251	20	-	-	-
Right Internal carotid artery	154	113	2	2	-
Left Internal carotid artery	157	112	-	1	1

**Table 3:** Characteristics of 271 patents undergoing elective CABG, Number of patients with plaque in carotid arteries categorized by grade of the peak systolic velocity.

	Peak systolic velocity		
	
	<125 _Cm/s_	125–230_Cm/s_	>230 _Cm/s_
Right common carotid artery	271	-	-
Left common carotid artery	269	2	-
Right Internal carotid artery	268	2	1
Left Internal carotid artery	268	2	1

Plaque formation in common carotid was not significantly different between two genders but the stenosis of left internal carotid was more frequently seen among men (p = 0.020) and the peak systolic flow and velocity was significantly lower (p = 0.0216).

Patients with plaques in right or left internal carotid arteries were significantly older (p = 0.007, p = 0.006) while no relation was found between age of the patients and presence of plaque in common carotids arteries.

## Discussion

The present study, showed a low incidence of severe carotid artery stenosis in Iranian patients undergoing elective CABG.

Complications of cardiac surgery have brought the carotid disease in to focus. Unlike other complications associated with CAB including hemorrhage, infection, and myocardial infarction, stroke represents an often irreversible, lifelong, and debilitating complication that frequently negates the benefits of coronary revascularization.

Several studies have evaluated the carotid disease as a risk factor for post operative CVA which deteriorates the quality of life and increases the mortality. Carotid endarterectomy has been recommended for symptomatic patients who have >50% narrowing of the ICA and for asymptomatic patients with 70% or greater narrowing of the ICA [15].

The prevalence of >50% carotid artery stenosis in patients undergoing CABG varies in different studies from 2% to 18% [16,17]. Berens et al, reported carotid stenosis >50% and >80% to be 17% and 5.9% respectively in elderly patients undergoing cardiac surgery while the prevalence of post operative CVA was 2% [18]. Rath et al, reported lower risk of CVA in patients with less than 70% stenosis of carotid or undergoing endarterectomy [19].

Salasidis et al, reported a higher (8.5%) prevalence of severe carotid stenosis in prekoperative assessment of patients undergoing CABG which were significantly older and showed a higher prevalence of CVA in this group (18.5% vs 7% in subjects with normal carotid) [20]. Gasera et al, showed the severe stenosis of carotid artery to be 12% in candidates of CABG [21].

In our study, significant internal carotid artery stenosis (>50%) in patients undergoing elective CABG was found in 1.8% of cases while only 1.1% had critical (>70%) stenosis. Thus, regardless to the high incidence of carotid artery disease in the study population, the prevalence of severe stenosis is lower than published western experiences.

Inverse effect of age on stroke is in agreement with the association between age and stroke in general population which may be as a reason of increased risk factors (e.g. atherosclerosis, hypertension and diabetes mellitus). Iranian patients seem to undergo elective CABG in younger age probably as a result of insufficient and delayed medical interventions.

Some investigators have identified risk factors for carotid disease that could be used for more selective carotid screening. These risk factors include age [22,23], carotid bruit and prior neurologic event [24,25], prior carotid surgery [24], peripheral vascular disease [24], hypertension, diabetes, and smoking [23]. But to date, there are no consensus criteria for selective screening to provide guidance for centers seeking to systematically optimize their carotid screening practices.

Selective carotid screening can be comparable with nonselective screening in detecting significant carotid disease and it is worth to assess this in further studies specially in different centers.

## Competing interests

The author(s) declare that they have no competing interests.

## Authors' contributions

MT conceived the study, participated in its design and carried out the duplex ultrasound

AA participated in the design of the study, participated in coordination and was involved in recruiting patients from Shaheed Madani Hospital.

MF participated in the study design and coordination

FK carried out the duplex ultrasound and clinical evaluation and helped to draft the manuscript

SF performed the statistical analysis, drafted the manuscript and participated in critical revising of the manuscript

All authors read and approved the final manuscript.
